# Focal Hypoperfusion on Baseline Perfusion-Weighted MRI and the Risk of Subsequent Cerebrovascular Events in Patients With TIA

**DOI:** 10.1212/WNL.0000000000213930

**Published:** 2025-07-24

**Authors:** Sofiya Shamailova, Luiz Dalla Vecchia, Elias Auer, Pasquale Castigliego, Victor Ziegler, Marc Fluri, Anna Boronylo, Moritz C. Kielkopf, Arsany Hakim, Adnan Mujanovic, Johannes Kaesmacher, Ava Leigh Liberman, Barbara Birner, Thomas R. Meinel, Mirjam Rachel Heldner, David Julian Seiffge, Thomas Horvath, Marcel Arnold, Urs Fischer, Simon Jung, Morin Beyeler, Philipp Bücke

**Affiliations:** 1 Department of Neurology, Inselspital, Bern University Hospital, and University of Bern, Switzerland;; 2 Graduate School of Health Science, University of Bern, Switzerland;; 3 Institute for Diagnostic and Interventional Neuroradiology, Inselspital, Bern University Hospital, and University of Bern, Switzerland; and; 4 Clinical and Translational Neuroscience Unit, Feil Family Brain and Mind Research Institute and Department of Neurology, Weill Cornell Medicine, New York, NY.

## Abstract

**Background and Objectives:**

While MRI is known to be crucial for TIA workup, the benefit of perfusion-weighted imaging (PWI) is underexplored. We aimed to assess the association between focal hypoperfusion on baseline PWI MRI and the long-term incidence of subsequent acute ischemic stroke (AIS) after TIA.

**Methods:**

Consecutive patients with TIA who underwent baseline PWI MRI as part of their emergency consultation between January 2015 and December 2019 were retrospectively identified. For study inclusion, both a time-based (symptom duration <24 hours) and an imaging-based (no signs of ischemia on diffusion-weighted imaging) TIA definition were applied. Long-term incidences of AIS after TIA were identified based on follow-up reports. Associations between focal hypoperfusion and subsequent AIS were assessed using Cox regression models adjusted for predefined predictors of stroke occurrence including symptomatic extracranial or intracranial stenosis. In subgroup analyses, we aimed to determine effects of focal hypoperfusion within vs outside the expected TIA territory, defined as a brain region potentially correlating with TIA symptoms.

**Results:**

Of 1,359 eligible patients with TIA, 1,075 with PWI MRI (79%) were included (median age 70 years, 46% female). Focal hypoperfusion was identified in 211 patients (20%); in 116 of 211 (55%), hypoperfusion occurred within the expected TIA territory. The median time from symptom onset to imaging was 233 minutes (interquartile range [IQR] 131–632) for patients with focal hypoperfusion vs 229 minutes [IQR 140–441] for patients without (*p* = 0.42). Focal hypoperfusion was associated with a higher incidence of AIS (adjusted hazard ratio [aHR] 2.13; 95% CI 1.19–3.80). While this was observed for focal hypoperfusion within the expected TIA territory (aHR 3.95; 95% CI 2.05–7.60), there was no such association in case of focal hypoperfusion outside the expected TIA territory (aHR 0.72; 95% CI 0.25–2.03).

**Discussion:**

Focal hypoperfusion on acute PWI MRI was found in 1 in 5 patients with TIA. It was associated with a higher incidence of AIS during long-term follow-up, especially when within the expected TIA territory. Further research is needed to clarify the predictive value of focal hypoperfusion in relation to the incidence of AIS after TIA and to explore potential therapeutic implications.

## Introduction

A TIA is diagnosed in approximately 2 of 1,000 persons each year.^1,2^ Within 5 years, approximately 10% of those patients with TIA will experience an acute ischemic stroke (AIS).^3^ Moreover, up to 10% of patients with AIS report TIA symptoms during the week before their AIS.^4^ The ABCD_2_ score has been suggested to stratify the individual risk of subsequent AIS after TIA.^5,6^ However, the score is limited by the omission of important risk factors of AIS such as extracranial or intracranial atherosclerosis or atrial fibrillation.^7,8^ A timely diagnosis supported by brain imaging findings—namely an absence of any sign of cerebral ischemia—is, therefore, essential.^9,10^

If available, MRI is preferred over CT because diffusion-weighted imaging (DWI) is more sensitive and specific for detecting acute cerebral ischemia in patients with transient neurologic symptoms.^11-13^ In addition, perfusion-weighted imaging (PWI) might be of diagnostic value in identifying patients with high-risk TIA.^14-16^ However, only a few studies have so far investigated associations between PWI and the occurrence of new cerebrovascular events after TIA.^17,18^ These studies were limited by sample size, the use of CT instead of MRI, a focus on radiologic outcomes, and short follow-up time.

The aim of this study was to assess a potential association between focal hypoperfusion on baseline PWI MRI and the incidence of subsequent AIS. We hypothesized that focal hypoperfusion would be associated with an increased risk of AIS after TIA.

## Methods

### Patient Population

We conducted a retrospective cohort study on consecutive patients evaluated for TIA at our comprehensive stroke center (Bern University Hospital, Bern, Switzerland) between January 2015 and December 2019. All patients with a reliable diagnosis of TIA that required DWI negativity on MRI were identified using the local part of the Swiss Stroke Registry (for hospitalized patients) and the emergency department information system (for outpatients) and assessed for eligibility. Patients without brain MRI and PWI at baseline were excluded. The remaining patients were included in this study, regardless of the time between symptom onset and MRI acquisition. Given that patients observed multiple events, only the index TIA was considered. At our institution, MRI including DWI and PWI sequences is the primary initial imaging modality for evaluating all patients with suspected AIS or TIA.^19^ In case of transient neurologic symptoms that occurred within 7 days before our notification, patients are examined by a senior neurologist in our emergency department immediately. We offer patients with symptoms noticed more than 7 days before our notification an emergency consultation on the following day, including MRI.

### TIA Definition

Patients with TIA were identified according to the definition used in the National Institute of Neurological Disorders and Stroke criteria (time-based definition with symptoms lasting <24 hours) and the Platelet-Oriented Inhibition in New TIA and Minor Ischemic Stroke Trial criteria (excluding patients with uncertainties regarding TIA diagnosis together with an additional focus on imaging [in our case, DWI negativity]).^20,21^ Adjudication was made by 3 junior neurologists (P.C., V.Z., M.F.), with discrepancies and uncertainties adjudicated by a senior vascular neurologist (P.B.). Patients were considered to have a reliable TIA diagnosis if they reported relevant clinical symptoms (monoparesis or hemiparesis, sensory disturbances in an entire limb or both ipsilateral limbs, aphasia, monocular loss of vision, homonymous hemianopia, or a combination of symptoms) and met both time-based (neurologic deficits reversible within 24 hours) and tissue-based (no signs of ischemia on baseline DWI) criteria.^22,23^ Patients with isolated double vision, isolated dizziness/vertigo, isolated dysarthria, transient loss of consciousness, and sensory disturbances affecting parts of a limb or the face were not included.

### Data Acquisition

Demographic and clinical characteristics were extracted from the local part of the Swiss Stroke Registry and the local electronic health records (EHRs; P.C., V.Z., M.F.). These included age at admission, sex, prestroke functional independence (defined as a modified Rankin Scale score ≤2), cerebrovascular risk factors (hypertension, diabetes mellitus type II, hyperlipidemia, history of smoking, previous stroke, TIA symptoms within 7 days before admission), imaging findings (e.g., symptomatic stenosis [≥50% extracranial or intracranial stenosis]), surgical or interventional treatment of symptomatic large artery stenosis, length of stay, and inpatient vs outpatient etiologic workup. The time between last known well and baseline brain imaging was calculated from the EHR. We used the ABCD_2_ score (0–7 points) as an ordinal variable, stratifying the risk of AIS after TIA as follows: 0–3 points for low, 4–5 points for moderate, and 6–7 points for high AIS risk.^5,6^ Because some patients underwent diagnostic workup in an outpatient setting, the Trial of ORG 10172 in Acute Stroke Treatment classification might be misleading and was, therefore, not reported.^24^ Details of secondary stroke prevention measures prescribed at discharge (antiplatelet drugs or anticoagulants) were extracted from the EHR. Antiplatelet drugs included a standard dose of aspirin (100 mg once daily), clopidogrel (75 mg once daily), or both (if indicated).

### Neuroimaging Analysis

All MRI studies were performed in our emergency department as part of the acute evaluation of patients with suspected AIS. Characteristics of MRI protocols (including DWI sequences, PWI sequences, and creation of perfusion maps) and MRI scanners in our institution have been described previously and are detailed in eMethods 1 and eTable 1.^25^

**Figure 1:**
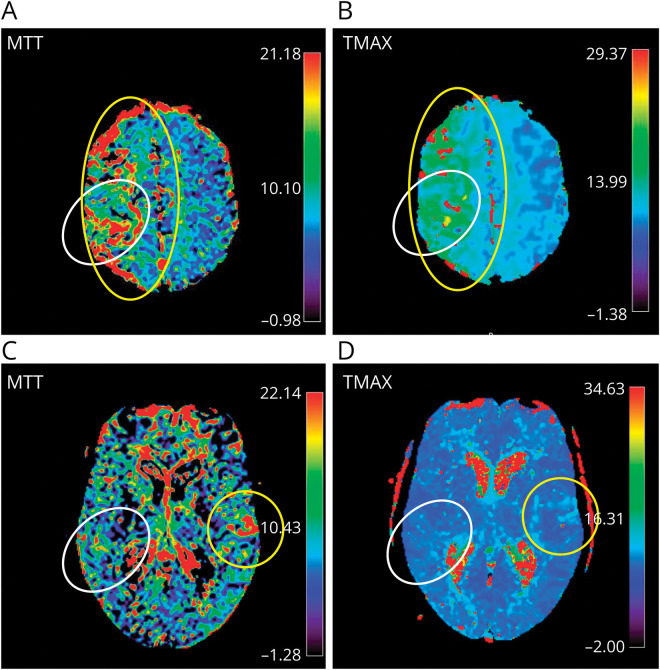
Assessment of Focal Hypoperfusion Within and Outside the Expected TIA Territory on Baseline Perfusion-Weighted Imaging MRI (A, B) Focal hypoperfusion within the expected TIA territory: PWI of a 71-year-old woman after 2 episodes of left facio-brachio-crural hemiparesis for 20 minutes with an expected TIA territory in the right middle cerebral artery territory (white circle). Focal hypoperfusion (yellow circle for perfusion delay) lies within the expected TIA territory in the right hemisphere and corresponds to prolonged MTT (up to 21 seconds, A) and prolonged Tmax (up to 15 seconds, B). TIA etiology was a symptomatic extracranial stenosis of the internal carotid artery. (C, D) Focal hypoperfusion outside the expected TIA territory: PWI of an 82-year-old woman after an episode of left-sided facial paresis for 20 minutes with the expected TIA area in the right hemisphere (white circle). There was no focal hypoperfusion within the expected TIA territory, but rather outside it (yellow circle), in the left opercular area, corresponding to slight prolongation of MTT (up to 20 seconds, C) and Tmax (up to 12 seconds, D). A specific TIA etiology could not be determined. MTT = mean transit time; PWI = perfusion-weighted imaging; Tmax = time-to-maximum.

Focal hypoperfusion was identified using the neuroradiologic report and by evaluating the PWI sequences in a 2-step semiautomated process.^25-28^ First, the DWI map was spatially coregistered with the time-to-maximum sequence from the same examination with a threshold of >6 seconds. In the second step, the mean transit time map was used as an overlay for the compound image from the first step to ensure comprehensive identification of hypoperfusion. Coregistering and overlaying were performed in ITK-SNAP (version 4.0, Penn Image Computing and Science Laboratory, University of Pennsylvania). Two experienced stroke neurologists (S.S., L.D.V.) blinded to EHR data assessed the presence of focal hypoperfusion on the coregistered and overlaid map. In a second reading after finalizing the database, focal hypoperfusion was classified as either within or outside the expected TIA territory according to TIA symptoms and lateralization (P.B., Figure 1). Expected territories for TIA symptoms were defined a priori (details in eMethods 2). Furthermore, the cause of hypoperfusion (vessel occlusion, extracranial stenosis, intracranial stenosis, or other [comprising chronic brain lesion, anatomical variants, undetermined]) was identified (eFigure 1).^29,30^ Chronic brain lesions were defined as previous ischemic stroke, intracerebral hemorrhage, or parenchymal damage after brain surgery (due to brain tumor or arteriovenous malformation, among others).

### Outcomes

The primary outcome was incidence of imaging-confirmed AIS during long-term follow-up. Secondary outcomes were the incidence of any ischemic cerebrovascular event (a composite of recurrent TIA and AIS) and overall mortality.

The incidence of cerebrovascular events was assessed using follow-up reports from the EHR and the Swiss Stroke Registry. The follow-up time was defined as the time between index TIA and the date of a subsequent cerebrovascular event (or last documented follow-up if no event was reported). For patients with no documented follow-up, the number of follow-up days was given as “0.5” in the case of an emergency department visit (corresponding to the time elapsed between admission, brain imaging, and subsequent emergency department discharge), or the number of days spent in hospital for inpatients. For survival analysis, the follow-up time was defined as the time between the index TIA and the date of death for deceased patients or—for surviving patients—up to the last update of the Swiss Population Register, which documents the inhabitants' vital status on a monthly basis.

### Statistical Analysis

Baseline characteristics were compared between (1) included and excluded patients, (2) patients with and without focal hypoperfusion, and (3) patients with focal hypoperfusion within and outside the expected TIA territory. Baseline characteristics were reported as frequency (percentage) for categorical variables and median (interquartile range [IQR]) for continuous variables. The Wilcoxon rank-sum test and the Fisher exact test were used to assess differences between groups for continuous and categorical variables, respectively. Cumulative time-to-outcome-event rates were reported using Kaplan-Meier curves. The log-rank test and multivariate Cox regression analysis were used to assess associations between presence of focal hypoperfusion and outcome events. A subgroup analysis assessing potential associations between focal hypoperfusion within the expected TIA territory (as opposed to patients with focal hypoperfusion outside the expected TIA territory and those without hypoperfusion) and outcome events was performed. To reduce confounding effects, we performed additional subgroup analyses excluding patients with hypoperfusion due to chronic brain lesions, and according to determined causes of focal hypoperfusion.

To minimize the risk of overfitting (due to the expected number of outcome events),^31,32^ our multivariate regression model included the following predefined covariates: sex, ABCD_2_ risk stratification classes (incorporating all ABCD_2_ items), any symptomatic stenosis (≥50% extracranial or intracranial artery stenosis), and anticoagulants prescribed for secondary prevention at discharge (due to a suspected increase in risk of subsequent AIS in case of atrial fibrillation). The covariates were selected regarding their association with cerebrovascular events after TIA (based on available literature).^5,6,29,30,33,34^

To account for variables associated with focal hypoperfusion (differences in baseline characteristics; *p* < 0.05), an extended regression model was developed (eMethods 3).

The proportional Schoenfeld residuals were used to test the hazard assumption. Adjusted hazard ratios (aHRs) were reported with their associated 95% CI. Statistical significance was defined as a *p* value of <0.05. All analyses were performed using Stata 17 (StataCorp LLC, College Station, TX) and R (version 3.6.0, R Core Team, Vienna, Austria).

### Standard Protocol Approvals, Registrations, and Patient Consents

The study was approved by the local ethics committee (project ID: 2022-01560; Kantonale Ethikkommission Bern). According to the ethics committee's decision, no study-specific written consent was required. In case of refusal of the internal general consent regarding “the further use of health data for research purposes,” patients were not included in this study. This represents approximately 5%–10% of all patients with AIS or TIA at our institution.

### Data Availability

Study data are available on reasonable request to the corresponding authors and after clearance by the local ethics committee. The Strengthening the Reporting of Observational studies in Epidemiology reporting guidelines were used to ensure the appropriate presentation of study findings.

## Results

### Study Population

**Figure 2:**
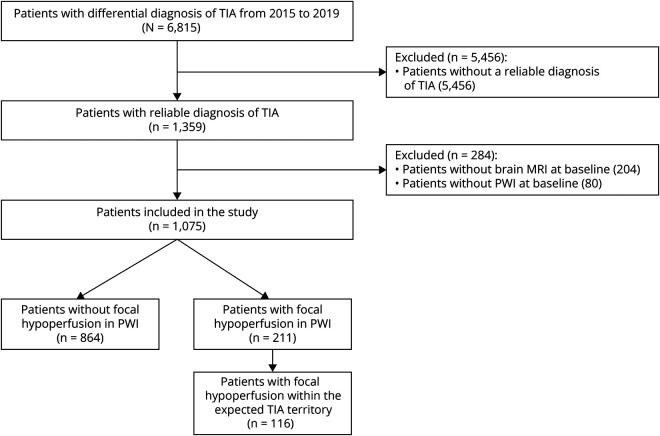
Study Flowchart Showing Inclusion and Exclusion of Patients PWI = perfusion-weighted imaging.

Between January 2015 and December 2019, 1,359 of the 6,815 patients with a suspected TIA seen at our emergency department had a reliable diagnosis of TIA and were assessed for eligibility (Figure 2). In total, 284 of 1,359 patients (21%) were excluded because of the unavailability of PWI MRI sequences at baseline. Of the 1,075 patients included in the study, focal hypoperfusion was identified in 211 (20%).

### Baseline Characteristics

Baseline characteristics of included and excluded (meaning no baseline PWI MRI) patients are summarized in eResults 1 and eTable 2. The total follow-up time for included patients was 1,448 patient-years for the primary outcome (incidence of AIS) and 3,743 patient-years for mortality (Swiss Population Registry).

There was no difference in the time from symptom onset to imaging in patients with vs without focal hypoperfusion (median 233 minutes [IQR 131–632] vs 229 minutes [IQR 140–441], *p* = 0.42; Table 1). Ninety-five percent of patients (n = 722/760) underwent imaging within 2 days of symptom onset. The maximum time between symptom onset and imaging was 21 days. Compared with patients without focal hypoperfusion, patients with focal hypoperfusion were older (74 years [IQR 62–82] vs 69 years [IQR 58–78], *p* = 0.01), more often reported TIA symptoms in the week before the index event (27% vs 12%, *p* < 0.001), and were more likely to have experienced previous strokes (27% vs 11%, *p* < 0.001). Moreover, patients with focal hypoperfusion demonstrated higher rates of cardiovascular risk factors and a higher rate of (any) symptomatic stenosis. Patients with focal hypoperfusion, compared with patients without hypoperfusion, were less likely to undergo outpatient etiologic workup (17% vs 30%, *p* < 0.001). There was no difference between the groups regarding secondary stroke prevention prescribed at discharge.

**Table 1:** Baseline Characteristics of Patients With and Without Focal Hypoperfusion in Perfusion-Weighted Imaging MRI After TIA

	Overall (N = 1,075)	No focal hypoperfusion (N = 864)	Focal hypoperfusion (N = 211)	*p* Value
Demographics, n (%)				
Sex, male	577/1,075 (47)	461/864 (53)	116/211 (55)	0.70
Age at admission, y, median (IQR)	70 (59–79)	69 (58–78)	74 (62–82)	<0.01
Independence before stroke (mRS score ≤2)	649/713 (91)	541/588 (92)	108/125 (87)	0.21
Medical history, n (%)				
Previous ischemic stroke	144/1,075 (13)	90/864 (11)	54/211 (27)	<0.001
TIA symptoms within 7 d before admission	157/1,075 (15)	103/864 (12)	54/211 (27)	<0.001
Hypertension	591/1,075 (55)	460/864 (55)	131/211 (65)	0.009
Diabetes mellitus II	134/1,075 (12)	97/864 (12)	37/211 (18)	0.014
Hyperlipidemia	525/1,075 (49)	405/864 (48)	120/211 (59)	0.005
Smoking history	214/1,075 (20)	173/864 (21)	41/211 (20)	1
TIA workup, n (%)				
Last known well to imaging, min, median (IQR)	229 (138–471)	229 (140–441)	233 (131–632)	0.42
Old infarcts (baseline MRI)	235/1,075 (22)	154/864 (18)	81/211 (39)	<0.001
Symptomatic stenosis				
Extracranial carotid stenosis	51/1,075 (5)	21/864 (2)	30/211 (14)	<0.001
Extracranial vertebral stenosis	3/1,075 (0.3)	3/864 (0.3)	0/211 (0)	
Intracranial stenosis	37/1,075 (3)	4/864 (0.5)	33/211 (16)	
Any symptomatic stenosis (composite)	91/1,075 (8)	28/864 (3)	63/211 (30)	<0.001
ABCD_2_ risk stratification				
Low risk (0–3 points)	313/1,075 (30)	263/864 (36)	50/211 (28)	0.083
Moderate risk (4–5 points)	461/1,075 (43)	358/864 (49)	103/211 (58)	
High risk (6–7 points)	138/1,075 (13)	113/364 (15)	25/211 (14)	
Length of stay, d, median (IQR)	1 (0.5–3)	1 (0.5–2.8)	2.6 (1–4.8)	<0.001
Outpatient etiologic workup	286/1,040 (28)	252/837 (30)	34/203 (17)	<0.001
Treatment, n (%)				
CAS/CEA	40/1,075 (4)	17/864 (3)	23/211 (14)	<0.001
Antiplatelet drugs (discharge)	861/1,075 (80)	699/864 (81)	162/211 (78)	0.18
Anticoagulation (discharge)	186/1,075 (17)	138/864 (16)	48/211 (23)	0.019
Long-term follow-up				
Follow-up (documented), n (%)	572/1,075 (53)	432/864 (50)	140/211 (66)	<0.001
Stroke during follow-up, n (%)	69/1,075 (6)	43/864 (5)	26/211 (12)	<0.001
Follow-up time for stroke, d, median (IQR)	22 (0.5–876)	5 (0.5–822)	137 (2–1,102)	<0.001
Cerebrovascular events during follow-up, n (%)	133/1,075 (12)	95/864 (11)	38/211 (18)	0.007
Follow-up time for cerebrovascular event, d, median (IQR)	11 (0.5–810)	4 (0.5–727)	147 (2–1,055)	<0.001
Long-term mortality rate, n (%)	177/1,075 (16)	127/864 (20)	50/211 (30)	0.006
Follow-up time for long-term mortality, d, median (IQR)	1,741 (1,269–2,263)	1,784 (1,307–2,328)	1,560 (1,092–2,070)	<0.001

Abbreviations: CAS = carotid artery stenting; CEA = carotid endarterectomy; IQR = interquartile range; mRS = modified Rankin Scale.

Categorical variables are listed as number/total number (percentage), and continuous or ordinal variables are listed as median (IQR).

Of the 211 patients with focal hypoperfusion, 116 of 211 (55%) had focal hypoperfusion within the expected TIA territory. When compared with patients with focal hypoperfusion outside the expected TIA territory (95/211, 45%), both groups had similar cardiovascular risk factors and did not differ regarding TIA symptoms (Table 2). The rate of any symptomatic stenosis was higher in patients with focal hypoperfusion within the expected TIA territory (56/116 [48%] patients vs 7/95 [7%] patients, *p* < 0.001).

**Table 2:** Baseline Characteristics of Patients With Focal Hypoperfusion Within and Outside the Expected TIA Territory

	Overall focal hypoperfusion (N = 211)	Hypoperfusion outside the expected TIA territory (N = 95)	Hypoperfusion within the expected TIA territory (N = 116)	*p* Value
Demographics				
Sex, male, n (%)	116/211 (55)	51/95 (54)	65/116 (56)	0.78
Age at admission, y, median (IQR)	74 (62–82)	75 (62–85)	73 (62–81)	0.17
Medical history, n (%)				
Previous ischemic stroke	53/211 (25)	26/95 (28)	28/116 (25)	0.63
TIA symptoms within 7 d before admission	54/211 (26)	23/95 (26)	31/116 (27)	0.87
Hypertension	131/211 (62)	54/95 (60)	77/116 (69)	0.24
Diabetes mellitus II	41/211 (17)	12/95 (13)	25/116 (22)	0.10
Hyperlipidemia	120/211 (57)	47/95 (52)	73/116 (65)	0.083
Smoking history	41/211 (19)	17/95 (19)	24/116 (22)	0.63
TIA symptoms, n (%)				
Amaurosis fugax	38/211 (18)	18/95 (20)	20/116 (18)	0.72
Hemiparesis	113/211 (51)	54/95 (59)	59/116 (52)	0.32
Hemihypesthesia	63/211 (30)	25/95 (28)	38/116 (33)	0.7
Speech disturbances	90/211 (43)	40/95 (44)	50/116 (44)	1
Vertigo	29/211 (14)	9/95 (10)	20/116 (18)	0.16
TIA workup, n (%)				
Last known well to imaging, min, median (IQR)	233 (131–632)	249 (146–663)	223 (128–549)	0.41
Old infarcts (baseline MRI)	81/211 (38)	37/95 (39)	44/116 (38)	0.89
Symptomatic stenosis				
Extracranial carotid stenosis	30/211 (14)	6/95 (6)	24/116 (21)	<0.001
Intracranial stenosis	33/211 (16)	1/95 (1)	32/116 (28)	
Any symptomatic stenosis (composite)	63/211 (30)	7/95 (7)	56/116 (48)	<0.001
Cause of focal hypoperfusion				
Vessel occlusion	28/211 (13)	5/95 (5)	23/116 (20)	<0.001
Extracranial carotid stenosis	31/211 (15)	7/95 (7)	24/116 (21)	
Intracranial stenosis	45/211 (21)	13/95 (14)	32/116 (28)	
Other	107/211 (51)	70/95 (74)	37/116 (32)	
ABCD_2_ risk stratification				
Low risk (0–3 points)	50/211 (24)	24/95 (28)	26/116 (28)	0.74
Moderate risk (4–5 points)	103/211 (49)	48/95 (56)	55/116 (60)	
High risk (6–7 points)	25/211 (12)	14/95 (16)	11/116 (12)	
Length of stay, d, median (IQR)	2 (1–5)	2 (0.7–4)	3 (1–5)	0.031
Outpatient etiologic workup	34/203 (17)	17/91 (19)	17/112 (15)	0.57
Treatment, n (%)				
CAS/CEA	23/211 (11)	4/95 (6)	19/116 (19)	0.012
Antiplatelet drugs (discharge)	162/211 (80)	66/95 (70)	96/116 (83)	0.033
Anticoagulation (discharge)	48/211 (23)	22/95 (24)	26/116 (23)	0.87
Long-term follow-up				
Follow-up (documented), n (%)	140/211 (66)	57/95 (60)	83/116 (72)	0.081
Follow-up time for stroke, d, median (IQR)	137 (2–1,064)	95 (1–1,127)	266 (4–1,101)	0.41
Stroke during follow-up, n (%)	26/211 (12)	6/95 (6)	20/116 (17)	0.02
Time to stroke, d, median (IQR)	74 (3–1,131)	793 (4–1,654)	18 (0.5–287)	0.16
Follow-up time for cerebrovascular event, d, median (IQR)	119 (2–997)	91 (1–1,055)	219 (3–1,044)	0.5
Cerebrovascular events during follow-up, n (%)	38/211 (18)	11/95 (12)	27/116 (23)	0.031
Time to cerebrovascular event, d, median (IQR)	94 (4–471)	122 (6–997)	52 (1–370)	0.37
Follow-up time for long-term mortality, d, median (IQR)	1,527 (1,091–2,000)	1,606 (1,127–2,002)	1,546 (1,091–2,114)	0.83
Long-term mortality rate, n (%)	50/211 (24)	25/95 (32)	25/116 (28)	0.61
Time to death, d, median (IQR)	762 (429–1,329)	678 (473–1,214)	938 (268–1,495)	0.46

Abbreviations: CAS = carotid artery stenting; CEA = carotid endarterectomy; IQR = interquartile range.

Categorical variables are listed as number/total number (percentage), and continuous or ordinal variables are listed as median (IQR).

### Primary Outcome

Compared with patients without focal hypoperfusion, patients with focal hypoperfusion had more documented follow-ups after hospital discharge (66% vs 50%, *p* < 0.001). During long-term follow-up, an AIS occurred in 26 of 211 patients with focal hypoperfusion (12%, 95% CI 7%–16%), vs 43 of 864 without focal hypoperfusion (5%, 95% CI 4%–7%) (*p* < 0.001).

**Figure 3:**
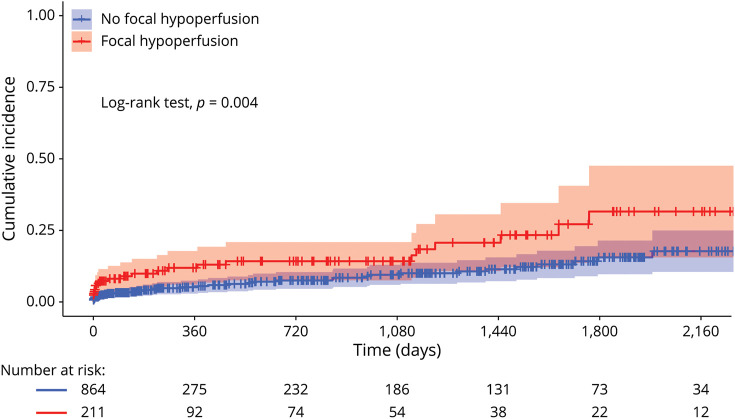
Long-Term Incidence of Acute Ischemic Stroke After TIA in Patients With and Without Focal Hypoperfusion on Baseline Perfusion-Weighted Imaging MRI Compared with patients without focal hypoperfusion (blue), patients with focal hypoperfusion (red) had a higher incidence of acute ischemic stroke after the index TIA (log-rank test, *p* = 0.004).

**Figure 4:**
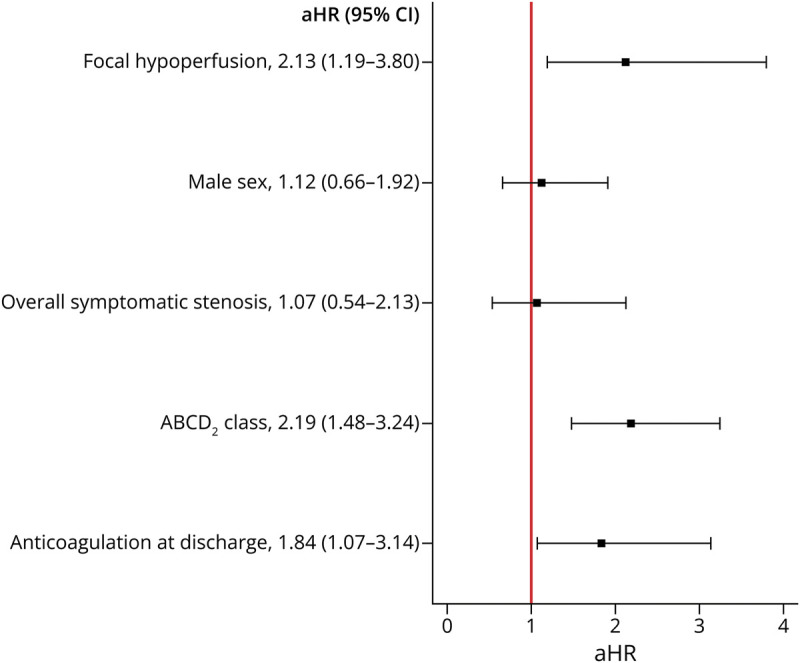
Multivariate Regression Model Assessing the Association Between Focal Hypoperfusion on Baseline Perfusion-Weighted Imaging MRI and Incidence of Acute Ischemic Stroke After TIA aHR = adjusted hazard ratio; AIS = acute ischemic stroke.

The cumulative incidence of primary outcome events in the long-term follow-up (shown in Figure 3) differed between groups (log-rank test *p* = 0.004). In the multivariate Cox regression model, focal hypoperfusion was associated with a higher incidence of AIS after TIA, compared with no hypoperfusion (aHR 2.13; 95% CI 1.19–3.80; *p* = 0.01, Figure 4).

In the extended model, this association remained unchanged (eResults 2, eFigure 2).

The median time (IQR) to AIS was 18 (0.5–287) days for patients with focal hypoperfusion within the expected TIA territory vs 793 (4–1,654) days for the remaining patients (*p* = 0.16).

The results were not affected when patients with focal hypoperfusion due to a chronic brain lesion were excluded (aHR 2.42; 95% CI 1.32–4.32; *p* = 0.004, eFigure 3). The incidence of AIS after TIA differed according to the identified cause of hypoperfusion (log-rank test *p* = 0.009). The cause of hypoperfusion in the presence of an AIS during follow-up was an occlusion in 31% of cases (n = 8/26), extracranial stenosis in 19% (n = 5/26), intracranial stenosis in 27% (n = 7/26), and other in 23% (n = 6/26). Owing to the low number of cases for each determined cause of hypoperfusion, adjusted analyses were not possible.

**Figure 5:**
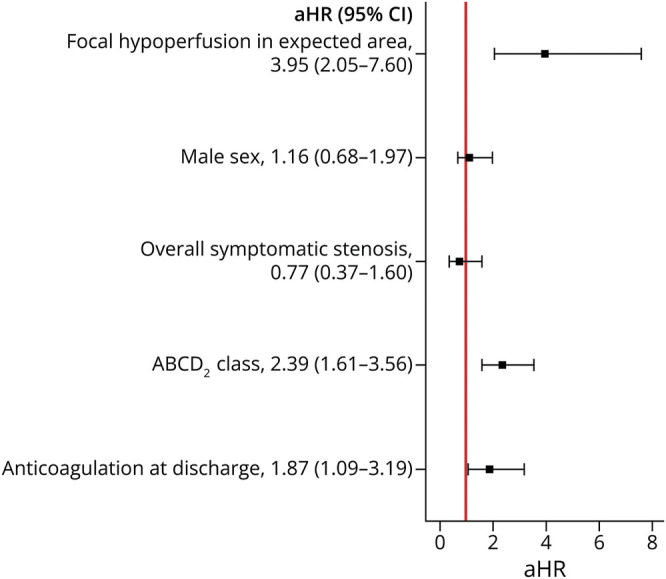
Multivariate Regression Model Studying the Association Between Focal Hypoperfusion Within the Expected TIA Territory on Baseline Perfusion-Weighted Imaging and Long-Term Incidence of AIS After TIA After adjustment, the presence of focal hypoperfusion within the expected TIA territory, compared with hypoperfusion outside the expected TIA territory or no hypoperfusion, was associated with a higher incidence of AIS in the long-term follow-up after TIA. Higher ABCD_2_ risk stratification classes and anticoagulation prescribed at discharge (as opposed to antiplatelet drugs) were also associated with a higher long-term incidence of AIS after TIA. aHR = adjusted hazard ratio; AIS = acute ischemic stroke.

Of the 26 patients with focal hypoperfusion and new AIS during follow-up, 77% had focal hypoperfusion within the expected TIA territory (n = 20). When compared with all other patients (with no hypoperfusion or hypoperfusion outside the expected TIA territory), the presence of focal hypoperfusion within the expected TIA territory remained associated with a higher incidence of AIS after TIA (aHR 3.95; 95% CI 2.05–7.60; *p* < 0.001, Figure 5). In the extended model, results remained unchanged (eFigure 4). Excluding patients with hypoperfusion due to chronic brain lesions did not affect the results (aHR 3.86; 95% CI 1.97–7.57; *p* < 0.001).

A perfusion deficit outside the expected TIA territory was not associated with higher incidences of subsequent AIS (aHR 0.72; 95% CI 0.25–2.03; *p* = 0.53). There was no difference in time between index TIA and occurrence of AIS in patients with hypoperfusion within vs outside the expected TIA territory.

### Secondary Outcomes

Cerebrovascular events after TIA occurred in 18% (95% CI 12%–21%) of patients with focal hypoperfusion (n = 38/211), vs 11% (95% CI 9%–14%) of those without focal hypoperfusion (n = 95/864, *p* = 0.007). There was no difference in the cumulative incidence of cerebrovascular events during long-term follow-up, as shown in eFigure 5 (log-rank test *p* = 0.20). After adjustment, these results did not change (aHR 1.23; 95% CI 0.77–1.98; *p* = 0.38).

Of the 38 patients with focal hypoperfusion who had cerebrovascular events during long-term follow-up, 71% had focal hypoperfusion within the expected TIA territory (n = 29). When compared with all other patients (with no hypoperfusion or hypoperfusion outside the expected TIA territory), the presence of focal hypoperfusion within the expected TIA territory was associated with a higher incidence of cerebrovascular events after TIA (aHR 2.19; 95% CI 1.27–3.78; *p* = 0.005, eFigure 6).

Overall, 278 patients were excluded from the analysis of long-term mortality because their long-term vital status was not available from the Swiss Population Register. Of the remaining patients, 30% (95% CI 24%–38%) of those with focal hypoperfusion (n = 50/165) died during long-term follow-up, compared with 20% (95% CI 17%–23%) of patients without hypoperfusion (n = 127/632) (log-rank test *p* = 0.002, eFigure 7). After adjustment, there was no difference between groups (aHR 1.12; 95% CI 0.72–1.74; *p* = 0.62, eFigure 8). The presence of focal hypoperfusion within the expected TIA territory was not associated with higher mortality rates (aHR 0.87; 95% CI 0.47–1.63; *p* = 0.67, eFigure 9).

## Discussion

Focal hypoperfusion on baseline PWI MRI was present in 1 in 5 patients with TIA, making this a relatively common finding. Our study suggests higher AIS occurrence rates in patients with focal hypoperfusion as detected on PWI MRI after TIA. This was attributable to a focal hypoperfusion within the expected TIA territory.

It is unclear whether perfusion-weighted sequences in patients with TIA provide additional value regarding identifying patients at risk of AIS. Owing to inconsistencies in TIA definitions (time-based, imaging-based), the interpretation of available data can be difficult.^17,18,26,28^ An association of CT perfusion abnormalities with early neurologic deterioration in patients with TIA has been described previously.^18^ Being CT-based, individual patients with AIS might have been misdiagnosed as having a TIA. Authors conducting MRI studies observed an increase in AIS after TIA in patients with focal hypoperfusion.^17,28^ Limitations included the focus on short-term radiologic outcomes and the inclusion of DWI-positive patients.^17,28^ According to recently adapted TIA definitions, patients with acute DWI lesions are considered those with ischemic stroke.^23,35^ Consequently, DWI positivity was an exclusion criterion in our MRI-based cohort.

Unlike global hypoperfusion patterns, focal hypoperfusion might be predictive of subsequent cerebrovascular events.^36,37^ In CT perfusion studies, the presence of focal hypoperfusion has been associated with factors such as higher NIH Stroke Scale scores, persisting symptoms, cerebral artery pathology/stenosis, and anatomical variants.^29,38,39^ Whether the observed hypoperfusion correlated with TIA symptoms was not reported.^17,18,28^ In our study, patients with any focal hypoperfusion on baseline PWI MRI were at a higher risk of AIS (12% vs 5% in patients without focal hypoperfusion).

We believe that focal hypoperfusion potentially responsible for TIA symptoms needs to be distinguished from (incidental) focal hypoperfusion of any cause. We, therefore, compared patients with focal hypoperfusion within vs outside the expected TIA territory. Focal hypoperfusion within the expected TIA territory, unlike focal hypoperfusion outside the expected TIA territory, was associated with subsequent AIS (aHR 3.95; 95% CI 2.05–7.60; *p* < 0.001). Clinically, alignment between symptom localization and focal hypoperfusion may support AIS risk stratification after TIA. However, this does not seem to apply when focal hypoperfusion occurs outside the expected TIA territory and, therefore, does not correspond to clinical symptoms. In 77% of patients with focal hypoperfusion who experienced AIS during follow-up, the initial hypoperfusion aligned with the expected TIA territory. Consistent with previously reported risk factors, we observed higher AIS incidences in patients with focal hypoperfusion patterns associated with vessel occlusion or intracranial and/or extracranial stenosis, compared with those with other focal hypoperfusion patterns, such as chronic brain lesions or anatomical variants.^34,40,41^ In our study population, mortality rates were not influenced by hypoperfusion patterns.

It is possible that the time between symptom onset and imaging could influence acute PWI findings. Therefore, it is noteworthy that symptom-to-imaging times in our cohort did not differ between patients with and without focal hypoperfusion. It is important to mention that, in case of chronic parenchymal brain lesions, cerebral perfusion parameters seem to remain abnormal over time.^42^ To minimize potential confounding, additional analyses excluding patients with focal hypoperfusion in the area of chronic brain lesions were performed. This subgroup analysis did not change the results of this study.

Current guidelines do not specifically recommend PWI MRI for TIA evaluation.^43,44^ This might change in the future because focal hypoperfusion within the expected TIA territory—irrespective of the presence of findings such as large artery atherosclerosis—might represent an additional risk factor of subsequent AIS in individual patients.

Our study has several limitations. First, owing to its retrospective design, all inherent biases apply. Therefore, we cannot exclude the possibility that patients might have been misdiagnosed leading to their accidental inclusion or removal from the analysis. Specific cerebrovascular risk factors (e.g., symptomatic extracranial or intracranial stenosis) might have been underreported because of the data acquisition process (affecting both patients with and without focal hypoperfusion). In addition, the number of patients with subsequent AIS might have been underestimated (in the entire study population) because individual cases might have been missed (follow-up was documented in approximately 50% of patients and higher in case of focal hypoperfusion). Nevertheless, the overall incidence of subsequent AIS is in line with previously published prospective data.^3,45^ Mortality rates should not have been affected by the percentage of documented follow-ups, given the separate data source of the Swiss Population Register that provides monthly updates. Second, the single-center design might limit generalizability. Finally, the definition of hypoperfusion on PWI MRI is another limitation. Although we applied frequently used thresholds, these are not universally accepted.^26-28^

In conclusion, focal hypoperfusion on PWI MRI seems to be associated with a higher incidence of AIS during long-term follow-up after TIA. This likely reflects focal hypoperfusion within the expected TIA territory, highlighting the importance of symptoms matching perfusion deficits in subsequent AIS risk stratification. By contrast, focal hypoperfusion outside the expected TIA territory was not associated with an increased risk of subsequent AIS. Further research is needed to clarify the predictive value of focal hypoperfusion in relation to the incidence of cerebrovascular events after TIA, assess its potential role as an independent risk factor, and explore potential therapeutic implications.

## Glossary

aHR: adjusted hazard ratio
AIS: acute ischemic stroke
DWI: diffusion-weighted imaging
EHR: electronic health record
IQR: interquartile range
PWI: perfusion-weighted imaging
